# Human-Induced Range Expansions Result in a Recent Hybrid Zone between Sister Species of Ducks

**DOI:** 10.3390/genes15060651

**Published:** 2024-05-21

**Authors:** Philip Lavretsky, Kevin J. Kraai, David Butler, James Morel, Jay A. VonBank, Joseph R. Marty, Vergie M. Musni, Daniel P. Collins

**Affiliations:** 1 Department of Biological Sciences, University of Texas at El Paso, El Paso, TX 79668, USA; vmmusni@utep.edu; 2 Waterfowl Program, Texas Parks and Wildlife Department, Canyon, TX 79015, USA; kevin.kraai@tpwd.texas.gov; 3 Central Coast Wetland Ecosystem Project, Texas Parks and Wildlife Department, Bay City, TX 77414, USA; david.butler@tpwd.texas.gov (D.B.); james.morel@tpwd.texas.gov (J.M.); 4 Northern Prairie Wildlife Research Center, U.S. Geological Survey, Jamestown, ND 58401, USA; jvonbank@usgs.gov; 5 Southwest Region—Texas Chenier Plain NWR Complex, U.S. Fish and Wildlife Service, Anahuac, TX 77514, USA; joseph_marty@fws.gov; 6 Southwest Region—Migratory Bird Program, U.S. Fish and Wildlife Service, Albuquerque, NM 87103, USA; dan_collins@fws.gov

**Keywords:** Anas, conservation genetics, ecology, hybridization, range expansion, south Texas Brush Country

## Abstract

Landscapes are consistently under pressure from human-induced ecological change, often resulting in shifting species distributions. For some species, changing the geographical breadth of their niche space results in matching range shifts to regions other than those in which they are formally found. In this study, we employ a population genomics approach to assess potential conservation issues arising from purported range expansions into the south Texas Brush Country of two sister species of ducks: mottled (*Anas fulvigula*) and Mexican (*Anas diazi*) ducks. Specifically, despite being non-migratory, both species are increasingly being recorded outside their formal ranges, with the northeastward and westward expansions of Mexican and mottled ducks, respectively, perhaps resulting in secondary contact today. We assessed genetic ancestry using thousands of autosomal loci across the ranges of both species, as well as sampled Mexican- and mottled-like ducks from across overlapping regions of south Texas. First, we confirm that both species are indeed expanding their ranges, with genetically pure Western Gulf Coast mottled ducks confirmed as far west as La Salle county, Texas, while Mexican ducks recorded across Texas counties near the USA–Mexico border. Importantly, the first confirmed Mexican × mottled duck hybrids were found in between these regions, which likely represents a recently established contact zone that is, on average, ~100 km wide. We posit that climate- and land use-associated changes, including coastal habitat degradation coupled with increases in artificial habitats in the interior regions of Texas, are facilitating these range expansions. Consequently, continued monitoring of this recent contact event can serve to understand species’ responses in the Anthropocene, but it can also be used to revise operational survey areas for mottled ducks.

## 1. Introduction

Biodiversity often has acute responses to human-induced ecological changes, ranging from local habitat loss to climate-level changes, thus resulting in local extirpation to complete species range shifts, respectively [1,2,3]. Among taxa, there has been mounting evidence that Aves continues to negatively respond to changing landscapes, with 30% of species experiencing declining populations and ranges over the last 50 years in North America [4]. However, not all species show decline but rather shifts, or even expansion, in their ranges in response to changing niche availability [5,6]. Importantly, it is essential to understand whether populations are truly declining or simply range shifting as these have differential and distinct management outcomes. Moreover, such range changes can result in increasing interactions between what were once allopatric species [7,8,9]. Consequently, genetic monitoring of range shifts not only provides an assessment of whether populations are genetically suffering due to declining populations, but it also more directly determines whether such events are potentially resulting in increasing interspecific hybridization; such assessments are becoming increasingly important for wildlife conservation in the Anthropocene (the Anthropocene currently has no formal status in the Divisions of Geologic Time https://pubs.usgs.gov/fs/2018/3054/fs20183054.pdf (accessed on 11 April 2024). It is used here to indicate a time when human activities have significant effects on the global environment) [10]. Here, we conduct a molecular assessment to test for and evaluate the outcomes of observationally noted population size and distributional shifts of two sister duck species, Mexican duck (*Anas diazi*) and mottled duck (*Anas fulvigula*), in parts of their range.

The Mexican and mottled duck are among 14 species that comprise the Mallard Complex, a group of ducks that evolved and radiated around the world over the last million years [11]. Recent genomic analyses have suggested that North America was first colonized by the mallard (*Anas platyrhynchos*) with glacial cycles over the last 500,000 years fragmenting expanding mallard populations and resulting in the eventual evolution of the American black duck (*Anas rubripes*), Mexican duck, and two mottled ducks in their respective regions [12,13,14,15,16,17]. Among these, Mexican and mottled ducks are the only non-migratory species, with the Mexican duck having adapted to the wetlands of the Chihuahuan and Sonoran Deserts, and the two populations of mottled ducks ranging across the freshwater and brackish marshes of Florida (i.e., FL mottled duck), as well as the Western Gulf Coast region spanning Alabama, Mississippi, Louisiana, Texas, and northeastern Mexico (i.e., WGC mottled duck). Note that genetic analyses have revealed that FL and WGC mottled ducks have been diverging in allopatry for some time given that they are genetically as distinct from one another as they are from the mallard [16,18]. Unfortunately, while mottled duck populations along the Texas coast have been drastically fluctuating between 50,000–100,000 individuals from 2011–2019, census counts have dramatically declined to ~29,000 in 2021, heightening continued conservation concern [19]. However, the Texas Parks and Wildlife Department (TPWD) has noted populations of putative WGC mottled ducks inhabiting the wetlands in the interior of Texas (i.e., south Texas Brush Country) with observed abundances nearly matching numbers that are being lost along the coast (K. Kraai personal observations). Thus, whether contemporary WGC mottled duck numbers are indeed declining, simply shifting geographically, or both remains unknown; but this information is required for future population management efforts. 

Although potential range shifts are only anecdotal for WGC mottled ducks, Mexican ducks have been expanding their geographical distribution and possibly abundance as they have been genetically confirmed west of their natural range of Mexico’s Interior Highlands as far as the coasts of Mexican states of Sonora and Sinaloa, and as far north as California [17,20]. Like the two mottled ducks, Mexican and WGC mottled ducks have been historically noted as also being naturally allopatric, with their respective restricted ranges separated by a zone of habitat in west Texas and eastern Mexico, which has thus far precluded extensive interbreeding [21]. However, it is in these intermediate regions that TPWD is noting increasing “dusky” ducks (i.e., Mexican- and/or mottled-like ducks) inhabiting wetlands in the Trans-Pecos and along the Rio Grande and in other available water corridors (K. Kraai *personal observations*). Additionally, eBird data have also shown increasing occurrences of these dusky ducks in the region over the last decade [22,23]. Thus, although less described, the Mexican duck may be similarly distributing itself eastward. Importantly, the geographical range extensions of Mexican and WGC mottled ducks are potentially resulting in secondary contact. Although empirical evidence of contemporary mottled × Mexican duck hybrids is limited, evolutionary models do support historical gene flow between Mexican and WGC mottled ducks [16,24]; thus, it is likely that these two taxa go through bouts of gene flow driven by range changes across time.

**Figure 1:**
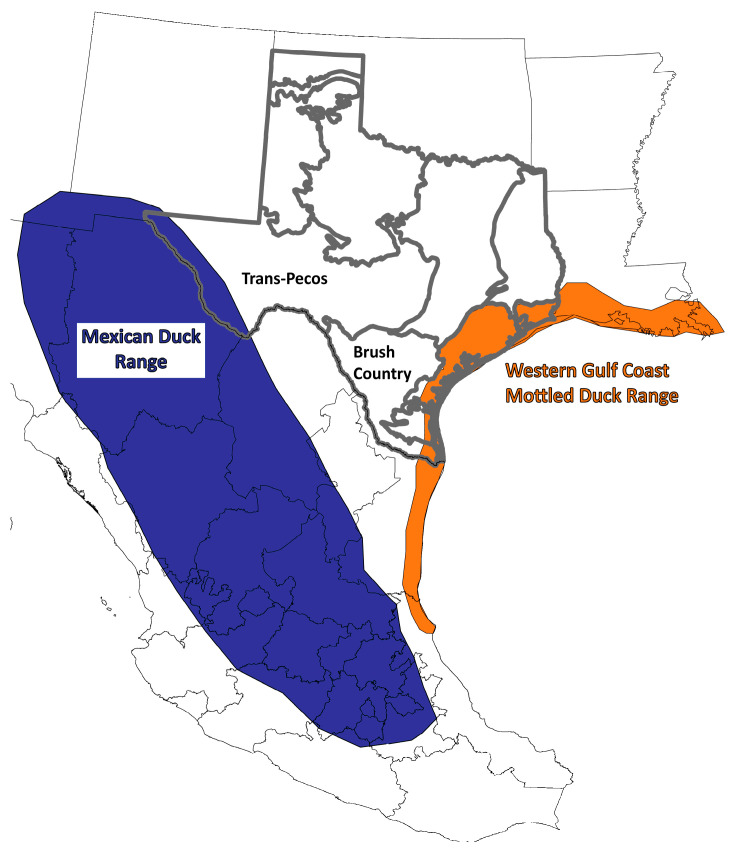
Map of the regions and endemic ranges of Western Gulf Coast mottled (Texas) and Mexican (Mexico) ducks as determined from the historical and contemporary accounts of Baldassarre [25] and eBird (www.ebird.org; accessed on 6 May 2024), respectively. Important study regions within Texas are denoted.

Phenotypic similarities between Mexican and WGC mottled ducks have not only made distinguishing between these monochromatic species difficult, but it has also made it difficult to even distinguish between their sexes [25]. Moreover, the phenotypic expression between their hybrids is yet to be described at all. Given that phenotypic identification remains largely unreliable, we aim to assess genetic ancestry using thousands of autosomal loci across samples representing both species’ ranges, including the south Texas Brush Country region, which the two are purportedly expanding into simultaneously (Figure 1). Doing so, we aim to determine whether Mexican and mottled ducks geographically overlap and, if so, whether they interbreed. We predict two possible scenarios: (1) a clear geographical separation between WGC mottled ducks and Mexican ducks with no overlap in the samples that are genetically determined to be mottled or Mexican duck, or (2) a zone of contact where samples genetically determined to be WGC mottled or Mexican duck geographically overlap with or without the presence of hybrids. Together, our study sheds light into how these species are responding to rapidly changing landscapes, including understanding the potential effects of secondary contact. Additionally, findings will be used to revise operational surveys to include any novel regions being used by expanding WGC mottled ducks when estimating population trends. 

## 2. Methods

### 2.1. Sample Collection and DNA Extraction

Blood or tissue was collected as part of state banding operations or were salvaged from hunters, respectively, from a total of 287 dusky ducks originating from Louisiana (*N* = 47), Texas (*N* = 185), and New Mexico (*N* = 55) between 2020–2023. DNA was extracted from all samples following DNeasy Tissue Kit protocols (Qiagen, Valencia, CA, USA). We visualized the presence of high molecular weight bands using gel electrophoresis with a 1% agarose gel.

### 2.2. Mitochondrial DNA Sequencing and Analyses

We used L78 and H774 primers to amplify via polymerase chain reaction (PCR) and Sanger sequence ~625 bp of the mitochondrial (mtDNA) control region [26,27] following the PCR reaction concentrations and thermocycler conditions described in Lavretsky et al. [11]. PCR products were visualized via agarose electrophoresis and then purified using ExoSAP-IT (ThermoFisher, Waltham, MA, USA). Clean PCR products were then sent for Sanger sequencing using the L78 primer on a 3130XL Genetic Analyzer at the University of Texas at El Paso, Border Biomedical Research Center’s Genomic Analysis Core Facility. The sequences were aligned and edited using Sequencher version 4.8 (Gene Codes). All sequences have been deposited in GenBank (accession numbers PP764859–PP765105; also see Supplementary Materials Table S1 for sample specific numbers). Note that mtDNA control region sequences for reference WGC mottled and Mexican ducks, as well as domestic and wild mallards from previous studies [12,16,18,20], were included in the alignments. The relationships among mtDNA haplotypes were assessed through a median-joining network constructed in POPART v 1.7 [28].

### 2.3. Nuclear DNA ddRADseq Library Preparation, Sequencing, and Bioinformatics

Double digest restriction-site-associated sequencing (ddRAD-seq) library preparation was conducted following the methodology of Lavretsky et al. [17] but with fragment size selection following Hernandez et al. (2021). Briefly, genomic DNA was enzymatically fragmented using SbfI and EcoRI restriction enzymes, followed by ligation of Illumina TruSeq compatible barcodes that permitted future de-multiplexing. Libraries were pooled in equimolar concentrations, and 150 base pair (bp) single-end (SE) sequencing was completed on an Illumina HiSeq X at Novogenetics Ltd. (Sacramento, CA, USA). Raw Illumina reads are deposited in NCBI’s Sequence Read Archive (SRA; BioProject PRJNA1108311; accession numbers SAMN41234194–SAMN41234409; also see Supplementary Materials Table S1 for sample specific numbers). 

We de-multiplexed the raw Illumina reads based on perfect barcode/index matches using the ddRADparser.py script of the BU ddRAD-seq pipeline (DaCosta and Sorenson 2014). As with mtDNA, previously published ddRAD raw sequence data generated using the same protocols were included in alignments and subsequent analyses, thus serving as reference WGC mottled duck [18], Mexican duck [17,20], wild mallard [12,15], and domestic mallards [14]. The domestic lineages included known game farm mallards and Khaki Campbells (*A. p. domesticus*), with the latter serving as a genetic signature of a “park” duck. All sequences were first trimmed or discarded for poor quality using Trimmomatic [29], and then remaining quality reads were aligned to a reference wild mallard genome [30] using the Burrows Wheeler Aligner v. 07.15 (bwa; [31]). Next, samples were sorted and indexed in SAMtools v. 1.6 [32] and combined using the “mpileup” function within bcftools (part of SAMtools v. 1.6) with the following parameters: “-c –A -Q 30 -q 30”. All steps through “mpileup” were automated using a custom in-house Python script (see Python scripts in [14]). Next, we used VCFtools v. 0.115 [33] to further filter aligned and genotyped VCF files for a minimum base-pair sequencing depth coverage of 5× (i.e., 10× per genotype), quality per base PHRED scores of ≥30, and any base-pair missing >5% of samples.

Though population structure analyses were based on autosomal loci only, the alignment of ddRAD-seq loci to the wild mallard reference genome also provided positions across sex chromosomes, allowing us to use differences in expected sequencing depth to molecularly assign sex across samples. In short, the heterogametic sex (i.e., female; ZW) is expected to have half the sequencing depth across both sex chromosomes when compared to autosome-linked loci, whereas the homogametic sex (i.e., males; ZZ) to show the same sequencing depth at the Z-sex chromosome and autosome-linked loci and no depth at W-sex chromosome linked loci. 

### 2.4. Nuclear Analyses

To evaluate nuclear population structure, we used autosomal ddRAD-seq bi-allelic single nucleotide polymorphisms (SNPs) only. Prior to our analyses, we used PLINK v. 1.90 [34] to further filter SNPs only found in a single individual (i.e., minimum allele frequency [maf] = 0.0016) and and/or those having >5% of samples missing. Additionally, we identified independent SNPs by conducting pair-wise linkage disequilibrium (LD) tests across ddRAD-seq autosomal SNPs (--indep-pairwise 2 1 0.5), in which 1 of 2 linked SNPs were randomly excluded if we obtained an LD correlation factor (*r*^2^) > 0.5. We conducted all analyses without a priori information on population or species identity. 

First, we used the PCA function in PLINK v. 1.90 [34] to perform a principal component analysis (PCA). Next, we used ADMIXTURE v. 1.3 to attain maximum likelihood estimates of population assignments across individuals, with datasets formatted for ADMIXTURE analyses using PLINK v. 1.90 [34], and following the steps outlined in Alexander et al. [35,36]. We ran each ADMIXTURE analysis with a 10-fold cross validation, incorporating a quasi-Newton algorithm to accelerate convergence [37]. Each analysis used a block relaxation algorithm for point estimation, and this was terminated once the change in the log-likelihood of the point estimations increased by <0.0001. Each analysis was run for *K* populations of 1 through 10, and the standard deviations were calculated under the optimum *K* population value based on 1000 bootstraps as implemented in the ADMIXTURE program. Although, the optimum *K* in each analysis was based on cross-validation errors, we examined additional values of *K* to test for further structural resolution across analyses. Finally, because previous research has showcased the difficulty that ADMIXTURE has when identifying subtle population structure as among Mexican ducks that follow an isolation-by-distance patterns [16,20], we coupled our inferences with co-ancestry estimates, as calculated in fineRADstructure [38]. In short, fineRADstructure infers a matrix of co-ancestry coefficients based on the distribution of identical or nearest neighbor haplotypes among samples, which emphasizes recent co-ancestry that is more efficient at recovering finer levels of structure [16,39]. We ran fineRADstructure with a burn-in of 100,000 iterations, which was followed by 100,000 Markov chain Monte Carlo iterations and subsequent tree building using default parameters. To visualize the results, we used R scripts provided with the program. 

Finally, we calculated nucleotide diversity (π) and pair-wise Weir- and Cockerham-weighted FST estimates for each genetic group recovered in population structure analyses, and which were based on 150 bp windows across ddRAD-seq autosomal loci and as implemented in VCFtools v. 0.115 [33].

## 3. Results

### 3.1. Mitochondrial DNA

**Figure 2:**
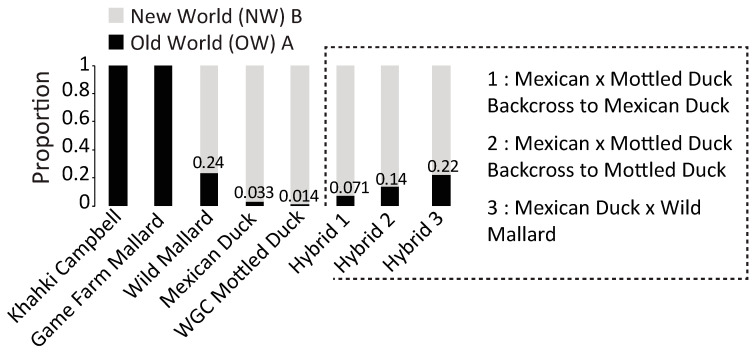
Proportion of the samples possessing Old World (OW) A versus New World (NW) B mitochondrial haplotypes among genetically vetted parental groups that included two domestic (Khaki Campbell (*N* = 13) and game farm mallards (*N* = 49)), three wild parental taxa (wild mallard (*N* = 102), Mexican duck (*N* = 246), Western Gulf Coast (WGC) mottled ducks (*N* = 146)), and three types of hybrids. The sample sizes for hybrid numbers 1, 2, and 3 are 28, 22, and 18, respectively (also see Supplementary Materials Table S1 for sample specific information).

A total of 596 overlapping bp of the mtDNA control region were sequenced across 624 samples (Supplementary Materials Table S1), with the haplotype network recovering known Old World (OW) A and New World (NW) B haplogroups (Supplementary Materials Figure S1). As expected, all the reference samples of domestic origins (e.g., Khaki Campbell’s and game farm mallards) carried OW A haplotypes, whereas the highest proportion of OW A mtDNA haplotypes in the wild groups was found among the wild mallards (34% of samples) and wild mallard backcross hybrids (22% of samples) (Figure 2). Conversely, we recovered very low rates of OW A mtDNA haplotypes among samples that were determined as genetically pure based on autosomal DNA (see below) Mexican (3.3% of samples) or mottled (1.4% of samples) duck.

### 3.2. Nuclear DNA

A total of 4851 ddRAD-seq autosomal loci (252,280 bp) met our criteria for sequencing coverage and missing data, with an average depth of 134 sequences/locus (depth range 16–246 sequences) across samples. In addition, we successfully assigned sex across the samples by plotting sequencing depth ratios between autosomal and recovered W- and Z-sex chromosome-linked ddRAD-seq loci (Supplementary Materials Figure S2).

**Figure 3:**
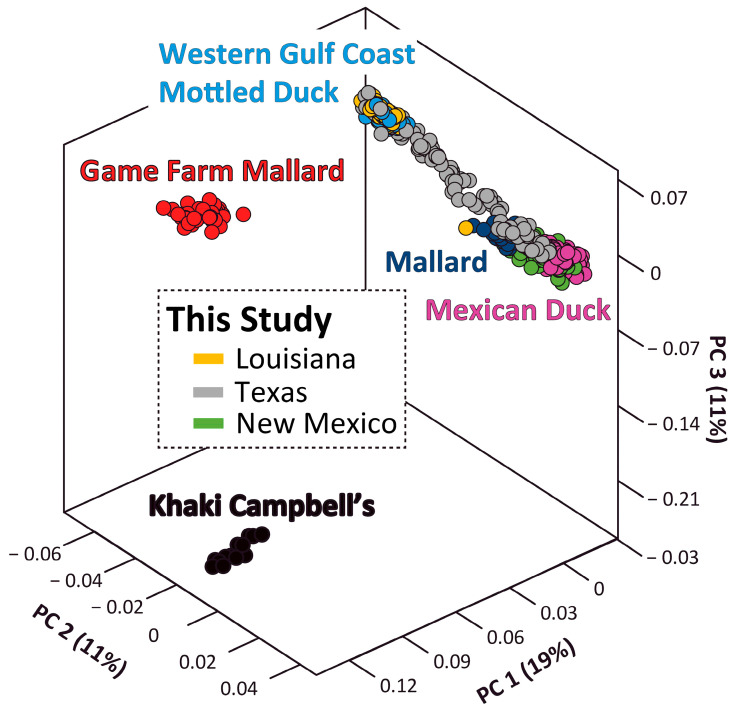
Plot of the first three principal components for the principal components analysis (PCA) of 19,165 independent bi-allelic autosomal single nucleotide polymorphisms (SNPs) that were sampled from reference domestic mallards, as well as wild mallards, Western Gulf Coast mottled ducks, and Mexican ducks. Our samples are color coded by sampling state, that includes Louisiana, Texas, and New Mexico.

**Figure 4:**
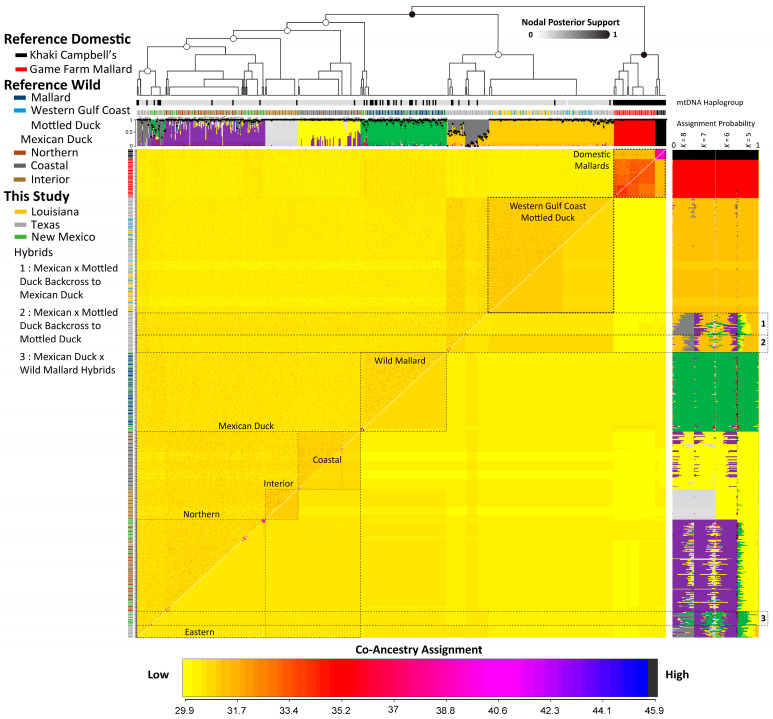
Estimated co-ancestry and individual assignment probabilities were based on 19,165 independent bi-allelic autosomal single nucleotide polymorphisms (SNPs) assayed across our samples from Louisiana, Texas, and New Mexico, as well as reference domestic mallards, wild mallards, Western Gulf Coast mottled ducks, and Mexican ducks. All samples are color-coded by population, including known Mexican duck subpopulations. In addition to providing ADMIXTURE individual assignment probabilities that were based on *K* population models 5–8 (right side), we overlayed respective standard errors on individual assignment probabilities based a *K* population model of eight (top). Finally, the bars below the cladogram denote whether the sample harbored Old World (OW) A (black) or New World (NW) B (gray) mitochondrial (mtDNA) haplotypes (Supplementary Materials Figure S1).

Population structure analyses were based on 19,165 (of 23,858) independent bi-allelic SNPs. Plotting the first three components of the PCA clearly differentiated feral Khaki Campbell’s, game farm mallards, WGC mottled ducks, and a group that included Mexican ducks and wild mallards (Figure 3). Further population structure resolution was achieved in ADMIXTURE analyses, with all reference samples assigned to their respective genetic groups under a population model of 6 (Figure 4). However, the known intraspecific structure among Mexican duck populations was only observed under *K* population values of 7 and 8. Specifically, we not only recovered known northern, interior, and west coast genetic clusters [17,20], but an eastern Mexican duck genetic cluster was also found when evaluating a *K* population model of eight. In fact, the alignment of individuals based on the fineRADstructure co-ancestry matrix showcased how the evaluation of population models that are less than seven resulted in perceived and variable mallard ancestry in ADMIXTURE individual assignments for these eastern Mexican ducks, but were lost when evaluating population models at seven and eight (Figure 4). The same was observed at a population model of five for Mexican ducks falling into the northern genetic group. However, in both cases, we did not find elevated levels of co-ancestry with wild mallards; thus, these samples were not hybrids but were a result of incomplete lineage sorting among groups of Mexican ducks harboring increased mallard ancestry (also see [20]). Conversely, groups of samples that were found to be intermediate in fineRADstructure co-ancestry also had generally similar ADMIXTURE assignment probabilities regardless of *K* population value. In short, by coupling ADMIXTURE individual and fineRADstructure co-ancestry assignments, we demarcated three genetic hybrid groups that included the following: (1) Mexican × WGC mottled duck hybrids that were backcrossed to Mexican duck (*N* = 28), (2) Mexican × WGC mottled duck hybrids backcrossed to mottled duck (*N* = 22), and (3) Mexican × wild mallard (*N* = 18) hybrids. Assuming a ≥10% interspecific assignment probability when evaluated under a *K* population of eight to be considered a hybrid, we recovered 20 of the 68 putative hybrids to be incongruent between analyses. Specifically, whereas a single Mexican × WGC mottled duck hybrid backcrossed to a WGC mottled duck sample (1/22; ~4.5%) was determined as a pure WGC mottled duck in ADMIXTURE analyses, five Mexican × wild mallard hybrids (5/18; ~28%) and 11 Mexican × WGC mottled duck hybrids backcrossed to Mexican duck (11/28; ~39%) were identified as pure Mexican duck. In all cases, we found increased co-ancestry assignments, suggesting some form of sibship, which is known to cause issues in ADMIXTURE analyses (i.e., violating the Hardy–Weinberg equilibrium) and may explain these incongruences. 

**Figure 5:**
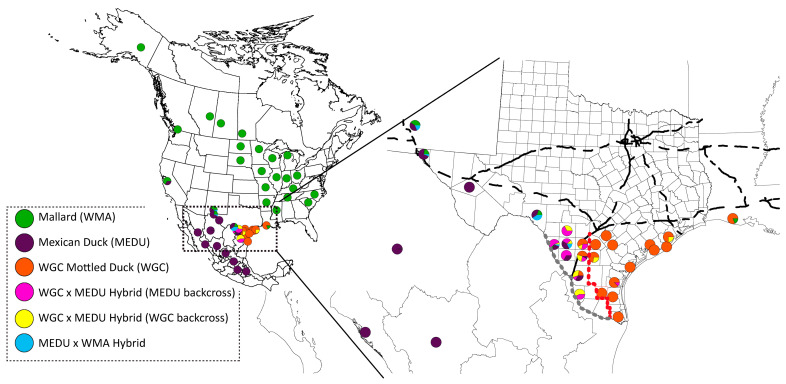
The proportion of each genetic group recovered in population structure analyses (Figure 4) represented geographically across the sampling locations, with each genetic group color-coded for reference. The expanded view includes sampling locations specific to this study across Louisiana, Texas, and New Mexico. Note that Texas includes county designations for reference purposes. Within Texas, the counties east of the dotted red line are regions where ≥90% of samples were genetically Western Gulf Coast (WGC) mottled ducks, and the ~100 km contact zone was defined by the region between the dotted gray and red lines.

Next, we geographically plotted per sample population assignments for the six possible populations, as determined from fineRADstructure clustering and ADMIXTURE assignment probabilities (Figure 5; Supplementary Materials Table S1). The plot recovers mallards, WGC mottled ducks, and Mexican ducks across their respective known ranges, as well as identifies the latter two in regions that were previously only observationally suggested (Figure 5). In short, we found genetically pure WGC mottled ducks as far west as La Salle county, Texas, and Mexican ducks as far east as Webb County, Texas. Most interestingly, both types of Mexican × mottled duck hybrids were recorded in between the regions of core Mexican duck and WGC mottled duck ranges, including generally shifting from WGC mottled duck to Mexican duck backcrosses when moving east to west. In general, 90% of Mexican × mottled duck hybrids could be found across a ~100 km area within the Brush Country region of south Texas, USA (Figure 5). Note that mallards and Mexican duck × mallard hybrids were generally found in the northern parts of Texas and southern New Mexico where the two are known to intermix, especially in urban settings [20]. 

Finally, through analyzing samples comprising parental groups (i.e., no hybrids), we found no significant difference in calculated nucleotide diversity between wild mallards, Mexican ducks, and WGC mottled ducks (Supplementary Materials Figure S3A). This translated to shallow estimates of relative divergences (Supplementary Materials Figure S3B), all of which were nearly identical to previous estimates between these species [16]. As expected, we found significant deviations in nucleotide diversity in both domestic breeds, with Khaki Campbell’s and game farm mallards having 50% and 25% reduction in genetic diversity, respectively, when compared to wild populations. Lost genetic diversity translated to elevated relative divergence estimates across pair-wise comparisons with Khaki Campbell’s (Avg. FST~0.20) and game farm mallards (Avg. FST~0.10). 

## 4. Discussion

Here, we provide empirical data showcasing how two sister species of ducks are simultaneously expanding their ranges. Specifically, we confirmed observations that Mexican and WGC mottled ducks are indeed expanding eastward and westward from their respective ranges, and which is resulting in a contact zone in the south Texas Brush Country where the two are evidently interbreeding (Figure 4 and Figure 5). Whereas relatively recent westward and northern range expansions have been confirmed molecularly for Mexican ducks [17,20], this is the first confirmation that they are also expanding eastward. Previous research of Mexican ducks residing in the Mexican states of Sonora and Sinaloa were found to be a result of a recent (~1990s) range extension via founder events of more interior populations from the state of Chihuahua, Mexico [13,20]. Thus, similar genetic signatures recovered here for the samples obtained from south Texas support the notion of a similar eastern range expansion via founder events of the same interior population of Chihuahua (Figure 4). Moreover, this study is the first molecular-based confirmation for the range expansion of WGC mottled ducks. Given the apparent range expansion of these ducks into Texas’ Brush Country region has only been observed since 2000, future research could improve our understanding regarding connectivity of these establishing populations with their respective sources via telemetry studies. For mottled ducks, testing whether declining populations along the Gulf Coast region are due to declining vital rates (i.e., survival and/or fecundity) and/or imbalanced emigration rates, as well as how and why mottled ducks are thriving in the largely semi-arid landscape of the south Texas Brush Country, will be critical for future management efforts for this regionally important species. The Mexican duck is perhaps the least known of all North American waterfowl, with their life cycle closely related to pluvial regimes and water availability [40]. However, both the western and the apparent eastern range expansions were predicted through recent genetic–environmental association-based niche modeling [20], suggesting they possess qualities that have been adapted as they evolved within the Chihuahuan desert, which may explain how they are generally expanding in all directions in ever-increasingly dry regions. 

### Conservation Considerations in the Anthropocene

Our study is an exemplar of some proximate species’ consequences of human-induced ecological change in the Anthropocene [1,2,3], with mottled and Mexican ducks responding via range expansions into novel landscapes and habitat types (Figure 5). However, we note that whereas Mexican ducks appear to be truly expanding without known population contractions elsewhere in their range [20], WGC mottled ducks may represent a distributional shift as they are declining in much of their natural coastal range [19]. We posit that climate-associated changes, including coastal habitat degradation coupled with increases in artificial habitat (e.g., human-made stock pond wetlands) in the interior regions of Texas, are facilitating the WGC mottled duck’s range shift. Specifically, while the brackish waters of Texas’ coast, where WGC mottled ducks are endemic, continue to degrade from sea level rise, pollution, etc. [41,42], various human enterprises (e.g., energy exploration, hunting preserves, etc.) in the more interior regions of Texas are causing the expansion of habitats that are desirable by waterfowl [43]. Thus, our findings suggest that WGC mottled ducks are responding to both human-mediated changes by leaving habitats that are declining in quality for habitats that are apparently increasingly expanding in abundance. Unlike the WGC mottled duck and many other bird species [4], the Mexican duck appears to be experiencing a range expansion in the Anthropocene. While the proximate cause(s) for this remain unknown, they too are responding to increasingly available habitats in the south Texas Brush Country and Trans-Pecos regions (Figure 5). 

Recent range changes in WGC mottled and Mexican ducks provide ideal systems to expand our understanding of how species are and will respond to the increasingly growing human footprints around the world. Toward this end, future work will need to first test between range expansion versus shifts for both species. For the WGC mottled duck, future research could focus on understanding the connectivity between coastal and interior habitats with the use of telemetry technology, along with the potential expansion of the April breeding surveys into areas of the south Texas Brush Country, where individuals can reliably be assumed to represent the species (i.e., ≥90% of individuals are mottled duck; see red line in Figure 5). Similarly, understanding the connections between the Mexican ducks of interior Mexico and their eastward expanding population will likely require the use of telemetry technology along with updated population estimates across their range. In short, a range expansion may be expected to maintain some individuals moving between source and founder location(s) in conjunction with limited population loss in the former. Conversely, the unidirectional movement of individuals along with continued population declines and increases in the source and founder locations, respectively, would be more representative of a range shift. Importantly, whereas the apparent range shift may be aiding WGC mottled ducks to escape deteriorating coastal environments, shifting into environments that could be maintained but are not optimum could in fact result in increased extinction risk to the species [44]. Moreover, the habitat into which WGC mottled and Mexican ducks are shifting is primarily unnatural itself and will require consideration when attempting to determine the region’s long-term sustainability for the species. Regardless, establishing vital rates (i.e., survival and fecundity) of the WGC mottled ducks residing in the south Texas Brush Country is important to understand the near- and long-term viability of the species in the region, potentially until coastal habitats can be revitalized if they are not lost [45]. We also acknowledge that, despite possessing a fraction of the census size of wild mallards (North American N_C_~7 million; [46]), Mexican (N_C_~55,000; [47,48]) and WGC mottled (N_C_~150,000; [19]) ducks carry equivalent genetic diversity (Supplementary Materials Figure S3A); a result of their recent ancestry and thus incomplete lineage sorting and not hybridization [16]. Consequently, we posit that the retention of such high genetic variation may indeed result in expanded adaptive qualities of one or both species, and this should continue to be monitored as their populations fluctuate and shift.

Human-induced species range shifts are indeed resulting in an increasing incidence of anthropogenic hybridization [8,12,49]. In addition to species’ ranges now including the artificial habitats of south Texas Brush Country, these range changes are resulting in a ~100 km secondary contact zone in the south Texas Brush Country (Figure 1) where the two species are interbreeding (Figure 5). Historically, the mallard was considered to be the primary instigator of introgressive hybridization for many of these mallard-like ducks; however, population genetic studies largely do not support this notion [50]. More recently, studies have confirmed that the release of the domesticated game farm mallard breed results in wide-spread hybridization with wild mallard populations worldwide [12,51], however, we find limited evidence of the same scenario among sampled Mexican and WGC mottled ducks (Figure 2, Figure 4 and Figure 5). Specifically, we found limited evidence of OW A mtDNA haplotypes (Figure 2 and Figure 4) and no confirmed game farm mallard hybrids based on autosomal DNA (Figure 4) among the sampled Mexican and WGC mottled ducks. While game farm mallards and their hybrids are known to be released and reside across Mexican and WGC mottled duck ranges, they do not appear to be interbreeding at the rates observed among wild mallards [12], which is consistent with the results of a molecular study of South Carolina’s mottled duck populations [52]. Moreover, although we were able to re-confirm Mexican duck × wild mallard hybrids in New Mexico and Trans-Pecos region of Texas [20], we did not find any WGC mottled duck × wild mallard hybrids despite wild mallards being known to winter in the coastal and interior regions of Texas [53]. Note that previous research has found some indication of feral mallard introgression among Mexican duck populations near urban settings [20], which were not included here. For the Mexican duck, we acknowledge their increasing northward range expansion will continue to bring them into closer proximity with populations of breeding mallards; thus, likely increasing chances for continued hybridization, which will require expanded and continued genetic monitoring. Conversely, mallards (wild or domestic) appear to be of little genetic threat to mottled ducks (also see [54]). Rather, we demonstrate that changing ranges are resulting in the interbreeding between WGC mottled and Mexican ducks (Figure 5). Importantly, recovering putative first generation (F1) hybrids alongside various stages of backcrosses suggests the absence of any pre- or post-zygotic reproductive barriers between these two closely related species of ducks. In fact, backcrossed hybrids predictably follow the geographical proximity to the range of the source parental population, which is moving eastward from more Mexican to WGC mottled duck-like (Figure 5). Whether the recorded secondary contact zone will remain static in the 100 km region (Figure 5), shift, or expand will require continued genetic monitoring coupled with the above demographic studies. 

## Data Availability

The mitochondrial DNA sequences were deposited in GenBank (accession numbers PP764859–PP765105). All raw Illumina sequences are available from the National Center for Biotechnology Information Sequence Read Archive (BioProject accession numbers PRJNA1108311). All the other source data (e.g., VCF files, ADMIXTURE input, fineRADstructure input, etc.) used for all the figures and tables are also available on Figshare (accession doi: 10.6084/m9.figshare.25761390).

## Supplementary Materials

The following supporting information can be downloaded at: https://www.mdpi.com/article/10.3390/genes15060651/s1, Figure S1: Mitochondrial haplotype network; Figure S2: Nuclear-based sex identification; Figure S3: Calculated nucleotide diversity and estimates of relative divergence; Table S1: Sample information.
