# Supplementary material for: Human-Induced Range Expansions Result in a Recent Hybrid Zone between Sister Species of Ducks
Source: Genes (Basel). 2024 May 21;15(6):651. doi: 10.3390/genes15060651 (PMC11202560; doi:10.3390/genes15060651)
Supplement: Supplementary file 1 [file genes-15-00651-s001.zip › genes-2986635-supplementary Figures.pdf]

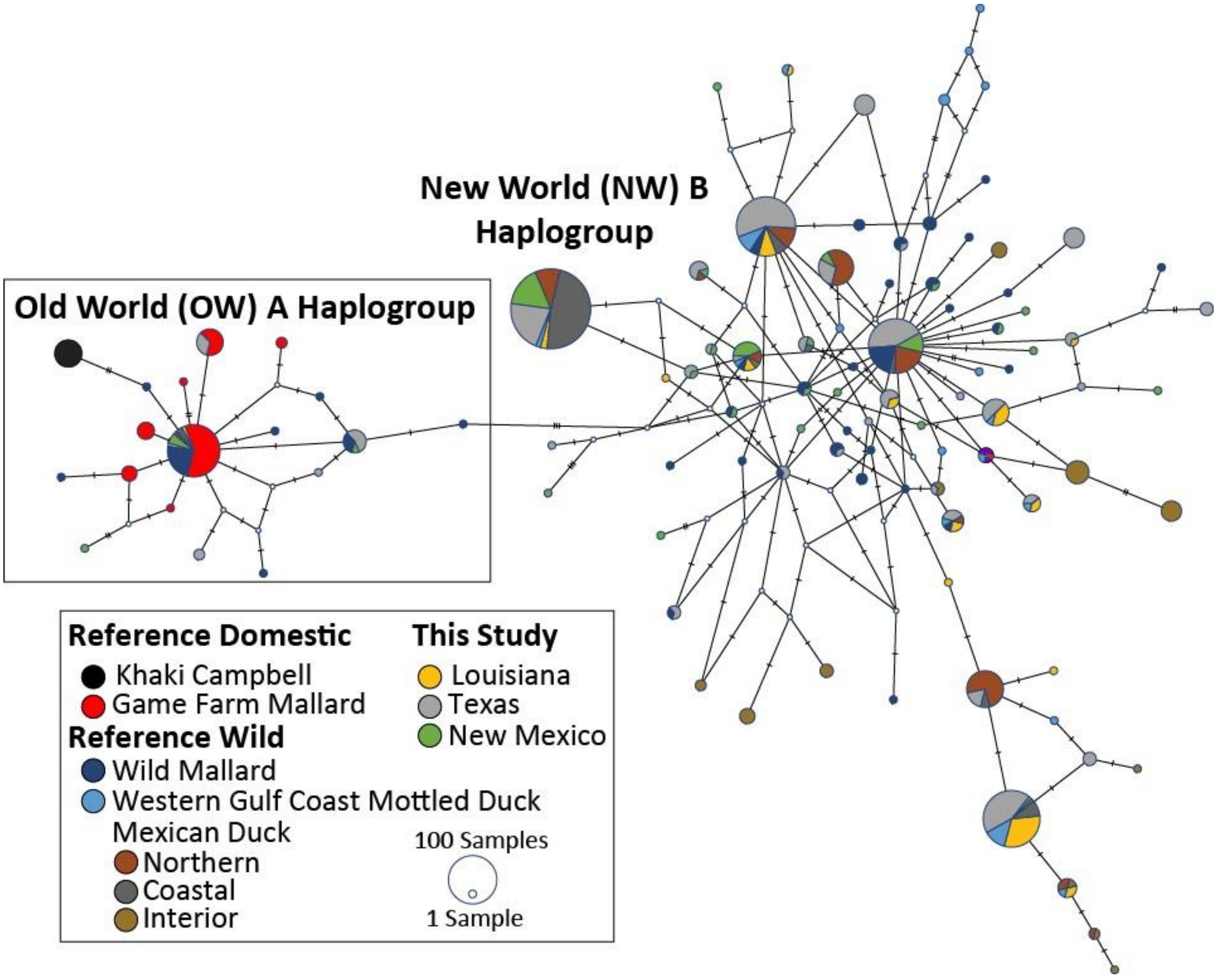


Figure S1. A haplotype network reconstructed from 596 base pairs of the mitochondrial control region (mtDNA) for sampled reference domestic mallards, as well as wild mallards, Western Gulf Coast mottled ducks, Mexican ducks, and samples obtained in this study from Louisiana, Texas, and New Mexico. Note that reference Mexican ducks are separated by known genetic clusters. Circles in the network denote different haplotypes with circle size proportionate to the number of samples represented within the haplotype, and the number of mutations separating two haplotypes equal the number of hash marks.

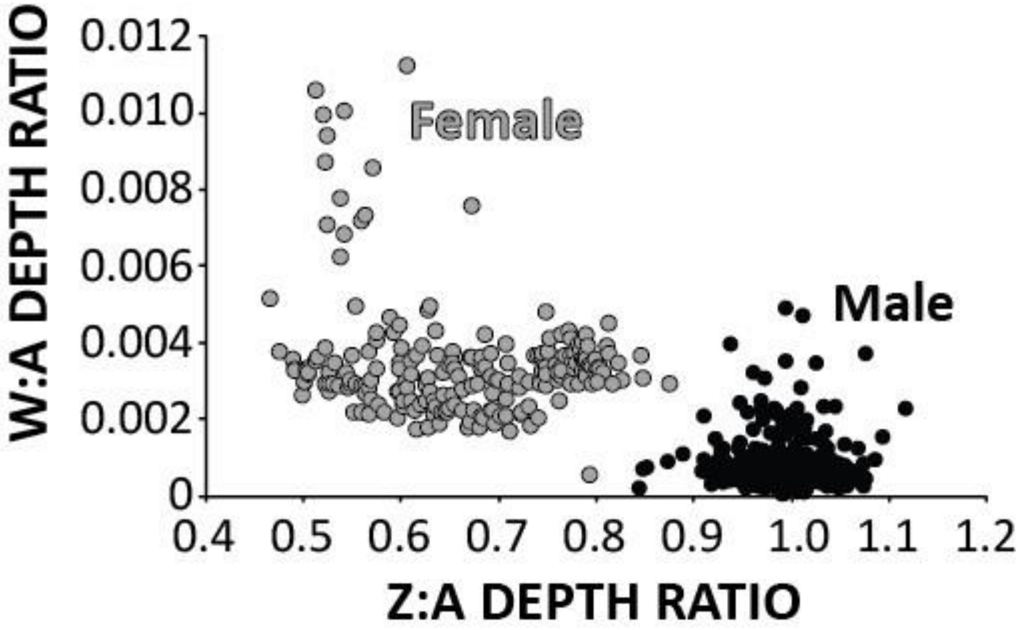


Figure S2. Plot of Z- or W-Sex chromosome versus autosomal (A) sequencing depth to identify the sex of sampled reference domestic mallards, as well as wild mallards, Western Gulf Coast mottled ducks, Mexican ducks, and samples obtained in this study from Louisiana, Texas, and New Mexico across the 2020-21 samples.

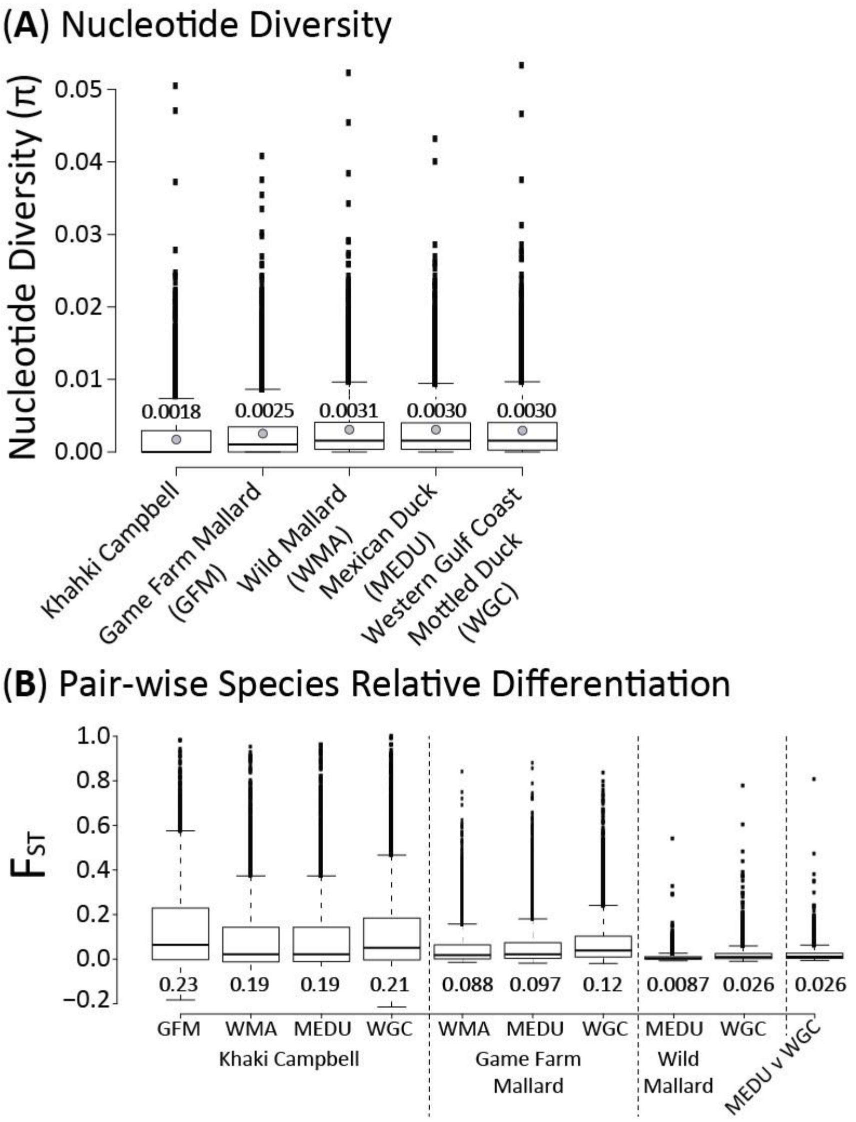


Figure S3. Ranges and respective averages of (A) calculated nucleotide diversity and (B) pair-wise population estimates of relative divergence for the five parental groups of ducks, and assessed across 4,851 ddRAD-seq autosomal loci.
